# The Possible Diagnostic and Prognostic Use of Systemic Chemokine Profiles in Clinical Medicine—The Experience in Acute Myeloid Leukemia from Disease Development and Diagnosis via Conventional Chemotherapy to Allogeneic Stem Cell Transplantation

**DOI:** 10.3390/toxins5020336

**Published:** 2013-02-18

**Authors:** Håkon Reikvam, Hanne Fredly, Astrid Olsnes Kittang, Øystein Bruserud

**Affiliations:** 1 Section for Hematology, Department of Medicine, Haukeland University Hospital, Bergen N-5021, Norway; E-Mails: hakon.reikvam@med.uib.no (H.R.); hanne.fredly@med.uib.no (H.F.); 2 Institute of Medicine, University of Bergen, Bergen N-5021, Norway; E-Mail: astrid.olsnes@med.uib.no

**Keywords:** acute myeloid leukemia, chemokines, systemic profiles

## Abstract

Chemokines are important regulators of many different biological processes, including (i) inflammation with activation and local recruitment of immunocompetent cells; (ii) angiogenesis as a part of inflammation or carcinogenesis; and (iii) as a bridge between the coagulation system and inflammation/immune activation. The systemic levels of various chemokines may therefore reflect local disease processes, and such variations may thereby be used in the routine clinical handling of patients. The experience from patients with myeloproliferative diseases, and especially patients with acute myeloid leukemia (AML), suggests that systemic plasma/serum cytokine profiles can be useful, both as a diagnostic tool and for prognostication of patients. However, cytokines/chemokines are released by a wide range of cells and are involved in a wide range of biological processes; the altered levels may therefore mainly reflect the strength and nature of the biological processes, and the optimal clinical use of chemokine/cytokine analyses may therefore require combination with organ-specific biomarkers. Chemokine levels are also altered by clinical procedures, therapeutic interventions and the general status of the patients. A careful standardization of sample collection is therefore important, and the interpretation of the observations will require that the overall clinical context is considered. Despite these limitations, we conclude that analysis of systemic chemokine/cytokine profiles can reflect important clinical characteristics and, therefore, is an important scientific tool that can be used as a part of future clinical studies to identify clinically relevant biomarkers.

## 1. Introduction

Chemokines are small proteins (8–12 kDa) [1]; approximately 50 human chemokines and 20 receptors have been identified (Table 1) [1,2,3,4,5,6,7,8,9,10,11,12,13,14,15,16,17,18,19,20,21,22,23,24,25,26,27,28,29,30,31,32], and they can be classified either on the basis of (i) the molecular structure, *i.e.*, the pattern of cysteine residues in the ligands or (ii) their functional characteristics, as inducible or inflammatory chemokines and constitutively expressed homeostatic chemokines [33]. Chemokines and their receptors are involved in the development of several disorders, including autoimmune diseases, cancer, as well as vascular diseases [34], and systemic (serum or plasma) chemokine levels may therefore serve as biomarkers for disease development or reflect disease activity, as well as treatment responses. 

The complexity of the chemokine system is seen at the receptor level, where especially inflammatory chemokines often bind several receptors and *vice versa* [34], and acute myeloid leukemia (AML) cells usually show constitutive release of many chemokines and express several chemokine receptors [20]. Furthermore, the chemokines are only a part of a complex network of interacting soluble mediators; during leukemogenesis, chemokines interact with other cytokines, especially hematopoietic growth factors and angioregulatory factors [35,36,37], but also with the matrix metalloprotease (MMP) system that seems to be directly involved in leukemogenesis and causes proteolytic cleavage and, thereby, activation of chemokines [38]. Serum/plasma chemokine profiles, therefore, have to be evaluated as a part of a more extensive network, both in studies of human AML [39,40] and probably also in studies of other human diseases [41,42]. The intention of this review is therefore (i) to review how systemic (serum/plasma) levels of individual chemokines are altered in AML, (ii) to describe and review how analysis of systemic profiles of soluble mediators, including chemokines, as well as their functionally interacting mediators, can be used for characterization, subclassification and prognostication of AML patients and (iii) to use AML as an example and, thereby, illustrate how analysis of systemic chemokine/cytokine profiles may become relevant for routine clinical handling of patients, *i.e.*, diagnostication, prognostication or treatment of patients.

**Table 1:** Chemokines and chemokine receptors [1,2,3,4,5,6,7,8,9,10,11,12,13,14,15,16,17,18,19,20,21,22,23,24,25,26,27,28,29,30,31,32].

Chemokine	Original name	Receptor	Releasing cells or organs	Important functions
**CXC (α) chemokine**				
CXCL1	GROα	CXCR2 > CXCR1, Duffy	MC, AML, EC, MP	↑ angiogenesis, anti-infectious activity
CXCL2	GROβ	CXCR2, Duffy	MC, MP	↑ angiogenesis
CXCL3	GROγ	CXCR2, Duffy	MC, MP	↑ angiogenesis
CXCL4	PF-4	CXCR3	Platelets, MK	Immunostimulatory, ↓ Angiogenesis
CXCL5	ENA-78	CXCR2	MC	↑ angiogenesis
CXCL6	GCP-2	CXCR1-2	MP, EC	↑ angiogenesis, immunostimulatory
CXCL7	NAP-2	CXCR1-2, Duffy	BMSC, MK	↑ angiogenesis
CXCL8	IL-8	CXCR1-2, Duffy	T-cells, MC, AML	↑ angiogenesis
CXCL9	MIG	CXCR3	T-cells, MC, AML	↓ hematopoiesis, ↓ angiogenesis
CXCL10	IP-10	CXCR3	AML, T-cells, DC	↓ Angiogenesis, involved transplant rejection
CXCL11	I-TAC	CXCR3, CXCR7	AML, T-cells, DC	↓ Angiogenesis, involved transplant rejection
CXCL12	SDF-1	CXCR4, CXCR7	SLO, BMSC	↓ Angiogenesis, anti-infectious activity
CXCL13	BCA-1	CXCR5	SLO, DC	↓ hematopoiesis, anti-infectious activity
CXCL14	BRAK	Unknown	Epithelial cells	Fibroblast growth factor
CXCL15	Lungkine	Unknown	Mucosal, endocrine org	↑ neutrophil migration, regulation of hematopoiesis
CXCL16	SR-PSOX	CXCR6	DC, MP	↓ hematopoiesis
**CC (β) chemokine**				
CCL1	I-309	CCR8	T-cells	Immunostimulatory, ↑ angiogenesis
CCL2	MCP-1	CCR2, CCR4, Duffy, D6	MC, AML, EC, DC	↓ hematopoiesis, ↑ angiogenesis
CCL3	MIP-1α	CCR1, CCR4-5, D6	EC, BMSC, T-cells, DC	↓ hematopoiesis, ↑ angiogenesis
CCL4	MIP-1β	CCR1, CCR5, CCR8, D6	T-cells, MC, AML	Regulation of inflammation
CCL5	RANTES	CCR1, CCR3-5, Duffy, D6	T-cells, MC, AML	Regulation of inflammation, tumor inhibition
CCL6		CCR1, CCR2-3, D6	BMSC	Regulation of inflammation
CCL7	MCP-3	CCR1-4, D6	MC	Regulation of inflammation, tumor inhibition
CCL8	MCP-2	CCR2-3, CCR5, D6	BMSC	Regulation of inflammation
CCL9/10		CCR1	SLO, MP	Regulation osteoclast function
**CC (β) chemokine**				
CCL1	I-309	CCR8	T-cells	Immunostimulatory, ↑ angiogenesis
CCL2	MCP-1	CCR2, CCR4, Duffy, D6	MC, AML, EC, DC	↓ hematopoiesis, ↑ angiogenesis
CCL3	MIP-1α	CCR1, CCR4-5, D6	EC, BMSC, T-cells, DC	↓ hematopoiesis, ↑ angiogenesis
CCL4	MIP-1β	CCR1, CCR5, CCR8, D6	T-cells, MC, AML	Regulation of inflammation
CCL5	RANTES	CCR1, CCR3-5, Duffy, D6	T-cells, MC, AML	Regulation of inflammation, tumor inhibition
CCL6		CCR1, CCR2-3, D6	BMSC	Regulation of inflammation
CCL7	MCP-3	CCR1-4, D6	MC	Regulation of inflammation, tumor inhibition
CCL8	MCP-2	CCR2-3, CCR5, D6	BMSC	Regulation of inflammation
CCL9/10		CCR1	SLO, MP	Regulation osteoclast function
CCL11	Eotaxin	CCR3, Duffy	MC	↑ angiogenesis
CCL12		CCR2, D6	Murine SLO	Regulation of inflammation, fibrosis
CCL13	MCP-4	CCR1-3, Duffy, D6	AML	Regulation of inflammation
CCL14	HCC-1	CCR1, CCR5, Duffy, D6	BMSC, SLO	Regulation of inflammation
CCL15	Lkn-1	CCR1, CCR3	T-cells, MC, DC	↑ angiogenesis, ↑ inflammation
CCL16	LEC	CCR1, CCR3, CCR5	Hepatocytes	↑ angiogenesis, immune regulating
CCL17	TARC	CCR4, D6	SLO, MC, AML, DC	Regulation of inflammation, graft rejection
CCL18	PARC	Unknown	SLO, DC	Anti-infectious activity
CCL19	ELC	CCR7, CCX-CKR	SLO, BMSC, DC	Regulation of AG presentation, cellular immunity
CCL20	LARC	CCR6	SLO	Regulation of inflammation, tumor inhibition
CCL21	SLC	CCR7, CXX-CKR	SLO, DC	Important for AG presentation, cellular immunity
CCL22	MDC	CCR4, D6	SLO, MC, AML, EC	Regulation of inflammation, graft rejection
CCL23	MPIF-1	CCR1	EC, MC, DC	↑ angiogenesis, biomarker inflammation
CCL24	MPIF-2	CCR3	AML	Allergic inflammation
CCL25	TECK	CCR9, CXX-CKR	Thymus	Involved in inflammation
CCL26	Eotaxin-3	CCR3	MC, EC	Allergic inflammation
CCL27	Eskine	CCR2-3, CCR10	Epidermal cells	Regulation of inflammation in the skin
CCL28	MEC	CCR10, CCR3	EC	Antimicrobial activity
**C (γ) chemokine**				
XCL1	Lymphotactin-α	XCL1	MP, Neutrophils	↑ hematopoiesis, anti-infectious activity
XCL2	Lymphotactin-β	XCL2	T-cells, MP	Regulation of immune-/inflammatory responses
**CX3C (δ) chemokine**				
CX3CL1	Fractalkine	CX3CL1	MP, MC, DC	↑ angiogenesis, atherosclerosis, inflammation

Abbreviations: SLO (secondary lymphoid organs), BMSC (bone marrow stromal cells), EC (endothelial cells), DC (dendritic cells), MP (macrophages), MC (monocytes), MK (megakaryocytes), AML (acute myeloid leukemia blasts) and AG (antigen).

**Table 2:** Serum chemokine levels in human AML.

Chemokine	Variations in Systemic Serum/Plasma Levels
CCL2	Untreated AML: Increased levels described in one study [43], but Fredly *et al*. [39], including mainly older patients above 65 years of age, as well as Kornblau *et al* . did not detect this difference [40].
	Expression in patient subsets: Fredly *et al* . [39] described decreased expression for elderly patients with CD14^+^ AML cells, whereas Kornblau *et al* . [40] described lower levels in younger patients with low-risk cytogenetic abnormalities.
CCL3	Untreated AML: Decreased levels described in one study [40], but normal levels described in another study, including mainly elderly patients [39].
CCL4	Untreated AML: Normal plasma levels [39,40].
CCL5	Untreated AML, patient subsets: Increased serum levels described in AML patients above 70 years of age compared with younger patients [39], and for the younger patients, levels were decreased compared with healthy controls [40].
CCL11	Untreated AML: Plasma levels are not generally altered [39,40]. Patient subsets: Decreased levels are seen for patients with high CD14 expression by the AML cells [39,40].
CCL17	Untreated AML: Decreased levels that show a further decrease during and following intensive chemotherapy [44].
CCL18	Untreated AML: Normal levels [45].
CXCL5	Untreated AML: Decreased levels [39].
CXCL8	Untreated AML: Increased serum levels are detected, and especially for patients with monocyte variants [39,46,47], these levels normalize when patients achieve complete hematological remission [47].
	Acute phase reactions: Increased levels are detected during febrile neutropenia and especially in septicemia or septic shock [48,49,50].
CXCL10	Untreated AML: Increased levels (most clearly seen in younger patients) have been detected [40,44]; these levels were not affected by chemotherapy, and increased levels persisted even after induction of hematological remission [51].
CXCL12	Untreated AML: Increased levels [43,44,52] and increased total CXCL12 levels are then accompanied by decreased levels of the functional non-cleaved form [52].

## 2. Systemic Levels of Single Soluble Mediators in Patients with AML: Chemokines *versus* Other Soluble Mediators

### 2.1. The Clinical Impact of Single Chemokine Levels

Even though AML cells show constitutive release of several chemokines [20], there is no general increase in the corresponding serum levels in untreated patients. The systemic levels of many chemokines have not been investigated, but the available results are summarized in Table 2. Generally, the levels of chemokine (C-C motif) ligand (CCL) chemokines did not differ (CCL3 in elderly patients, CCL4, CCL11, CCL18) or were decreased (CCL3 and CCL5 in younger patients, CCL17) when compared with normal healthy individuals, the only two exceptions being CCL5 that showed increased levels in elderly patients and possibly CCL2 that showed increased levels in one out of three studies [40,43,45]. In contrast, chemokine (C-X-C motif) ligand (CXCL) chemokines were usually increased (CXCL8, CXCL10, CXCL12) with only CXCL5 being decreased in human AML [39,40,43,44,46,47,48,49,50,51,52]. Cytogenetic abnormalities of the AML cells seem to have a minor influence on systemic chemokine levels, with only CCL2 showing an association with cytogenetic abnormalities (Table 2), whereas monocytic differentiation has been associated with the profile CCL2^low^CCL5^low^CXCL8^high^ [39]. The CCL2 levels were then relatively low in patients with favorable cytogenetics, whereas CCL2 levels were 5- and 6.67-fold increased for patients with intermediate and unfavorable cytogenetics, respectively. Associations between chemokine serum levels and cytogenetics have not been described in other studies. However, AML is very heterogeneous with regard to cytogenetic abnormalities, and the other studies generally included relatively few patients; and for this reason, detailed analyses of possible associations between cytokine levels and specific cytogenetic abnormalities were not possible. Platelet-derived growth factor-BB (PDGF-BB) showed a similar, but weaker association, whereas IL7 levels were lowest in the intermediate group, slightly increased in the unfavorable group and were highest in patients with unfavorable cytogenetics. The other cytokines showed no association with leukemia-associated cytogenetic abnormalities. Taken together, these observations suggest that the cytogenetic abnormalities have only a minor influence on systemic serum cytokine levels (and probably also cytokine profiles), including chemokine levels. Finally, Feng *et al*. [53] investigated systemic levels for 33 cytokines and confirmed that age-dependent differences in serum cytokine/chemokine levels may occur, even though this is relatively uncommon for healthy individuals’ tumor necrosis factor (TNF)α and patients with nonmalignant aplastic anemia (CXCL5), but not for patients with preleukemic myelodysplastic syndromes (MDS). As can be seen from Table 2, age-dependent differences may also occur in AML (CCL3, CCL5).

There are discrepancies between various studies with regard to systemic chemokine levels in untreated AML, *i.e.*, the studies by Fredly *et al*. [39] and Kornblau *et al*. [40]. The most striking difference between these two studies is that Kornblau *et al*. [40] included patients that were relatively young and fit for intensive chemotherapy, whereas Fredly *et al*. [39] included mainly elderly patients, with many of them being unfit for intensive therapy due to complicating diseases and poor performance status. 

Primary human AML cells usually show high constitutive release of CCL2/3/4/5, CXCL1 and CXCL8; a large subset of patients also shows relatively high release of CCL5/13/17/22/24 and CXCL5/9/10/11 [40]. Thus, high constitutive release is not necessarily associated with increased systemic chemokine levels, and this may at least partly be explained by the fact that the systemic levels are determined by a balance between release and binding/degradation. This observation also illustrates that systemic cytokine levels do not necessarily reflect the local bone marrow cytokine network.

The association between pretherapy chemokine serum levels and survival after intensive chemotherapy was investigated by Kornblau *et al*. [40]. These authors described an association between good prognosis (prolonged survival) and high serum levels of CCL5 and low CCL2 levels, the strongest association then being to CCL5. Several interleukins (high levels of IL2, IL4, IL5, IL10; low levels of TNFα) also showed an association with favorable prognosis and higher remission rates.

This influence of many different factors on systemic chemokine levels (e.g., age, AML cell differentiation, cytogenetics, possibly performance status and other diseases) will probably limit the use of single chemokine levels as biomarkers in AML patients, and analyses of chemokine/cytokine profiles may then be more reliable. 

### 2.2. Systemic (Serum/Plasma) Levels of Immunoregulatory Cytokines and Growth Factors Other than the Chemokines

Systemic levels of several other cytokines have also been compared for AML patients and healthy controls [41,54], and the levels of Interleukin-3 (IL3), IL6, thrombopoietin (Tpo) tumor necrosis factor TNFα and stem cell factor (SCF, c-kit ligand) seem to be increased in patients with untreated AML [47,49,55,56,57,58]. This overexpression will often decline after chemotherapy when disease control with complete hematological remission is achieved [47], although this is not always true (e.g., CXCL10, as described above, Tpo, SCF) [59]. Serum levels of single cytokines may also be associated with prognosis:

- Serum cytokine levels can also be altered by disease progression in patients with myeloproliferative neoplasms; IL2, soluble IL2 receptor and IL6 levels were increased after progression to AML in patients with progression from chronic myelofibrosis to AML [60].
- AML patients with high serum levels of hepatocyte growth factor (HGF) [61], TNFα [58] or vascular endothelial growth factor (VEGF) [62] seem to have decreased long-time survival after intensive chemotherapy.
- Several hematopoietic growth factors (*i.e.*, granulocyte colony-stimulating factor (G-CSF), granulocyte-macrophage colony-stimulating factor (GM-CSF), IL3, fms-like tyrosine kinase 3 ligand (Flt3-L)) show detectable levels in AML patients; these levels can be decreased by response to chemotherapy and/or increased by severe complicating infections [63].

### 2.3. Soluble Adhesion Molecules and Matrix Metalloproteases (MMPs) in Human AML

Several cellular adhesion molecules also exist in biologically active soluble forms, both in healthy individuals and AML patients; this is true both for the L/E/P-selectins, Intercellular adhesion molecule-1 (ICAM-1) and endocan [49,64,65]. These molecules are also important for cell trafficking, and the systemic levels of their soluble forms can be altered in response to intensive chemotherapy or complicating infections in AML patients. Furthermore, MMPs and the tissue inhibitors of matrix proteases (TIMPs) are probably also important in leukemogenesis or for chemosensitivity in human AML [66]. This role of the MMP/TIMP system may not necessarily be caused by their proteolytic activity and degradation of extracellular matrix; the TIMPs seem to have additional signaling functions in addition to their inhibitory effects on proteases (for references and a more detailed discussion, see [66]), and certain MMPs are in addition important for cleavage and, thereby, activation of chemokines [38]. 

### 2.4. Conclusion: Systemic Chemokine Levels as Biomarkers in Human Diseases Will Probably Be Most Useful when Investigated as Chemokine Profiles and Combined with Analysis of Other Biologically Interacting Soluble Mediators

Taken together, these studies of single chemokines suggest that their systemic levels reflect important disease characteristics with regard to disease development and chemosensitivity. However, the results are partly conflicting, probably because single chemokine levels are also affected by many different factors; the use of single chemokines as biomarkers will therefore be difficult, and analysis of chemokine profiles may be most relevant. It may then be relevant also to include analysis of other soluble mediators (*i.e.*, other cytokines, soluble adhesion molecules, extracellular enzymes) together with the chemokines (see summary in Table 3), because all these mediators form a functionally interacting network in regulation of proliferation, viability and trafficking for a wide variety of cells. 

## 3. Cytokine Classification Based on the Main Function in Human AML

The interleukins were originally defined as a separate entity based on their release by and effects on leukocytes, whereas the chemokines were identified as soluble mediators with important effects on cell migration (*i.e.*, chemotaxis) and with CXCL8/IL8 having a double classification. The chemokines were further subclassified either based on their molecular structure or their functional characteristics. These two examples illustrate that a simple classification of all cytokines is difficult. In our previous studies of the cytokine network in human AML, we therefore used a disease-dependent cytokine classification, *i.e.*, the main functions of a particular cytokine in the context of this particular disease are included as additional criteria for classification. We have then classified the cytokines into chemokines, as described in detail in Table 1, interleukins, growth factors and immunoregulatory cytokines (Table 4) [67,68,69]. However, it should be emphasized that such a simple classification does not reflect all the complex functions of a single cytokine in AML, and some cytokines have effects that could have justified classification into more than one subset, e.g., TNFα being a regulator of both immune reactions and hematopoiesis, certain chemokines acting both as AML growth factors and immunoregulators and VEGF acting both as a angioregulator and a directly-acting growth factor for AML cells. The use of such disease-dependent classifications may also become useful in the studies of chemokine/cytokine network in other diseases and not only in AML.

**Table 3:** A summary of soluble mediators interacting with the chemokine system [20,38,39,41,42,43,44,46,47,48,49,50,51,52,54,55,56,57,58,59,61,62,63,65,66,67,70].

Soluble mediators	Functional interaction
Hematopoietic growth factors	Several hematopoietic growth factors facilitate AML cell proliferation, including G-CSF, GM-CSF, M-CSF, IL1, IL3, SCF, Flt3-L [20,41,47,50,55,56,57,58,63,71,72].
Angioregulatory cytokines	Angiogenesis seems to be important, both for leukemogenesis and chemosensitivity and several angioregulatory cytokines interact with the pro- and anti-angiogenic chemokines [20,42,43,44,46,51,58,61,62].
Soluble adhesion molecules	Several adhesion molecules exist in biologically active soluble forms [49]. These molecules can be formed either by shedding from the cell membrane, or they are synthesized as soluble isoforms of the molecules; the molecules can interact with cell trafficking/migration [49,65].
Soluble cytokine receptors	Several cytokine receptors are also released in biologically active soluble forms, e.g., TNF and IL2 receptors [48,52]. The systemic levels of certain receptors have prognostic impact; the mechanisms behind this could be either competition for cytokine binding sites with the membrane-expressed receptors, transport of the cytokines or prevention of degradation [54].
Heat shock proteins	The chaperones can be released together with their client proteins. The soluble levels of certain heat shock proteins can have a prognostic impact in human AML, and they may facilitate presentation of cancer-associated antigens [39,70].
Matrix metalloproteases	MMPs and the inhibitory TIMPs regulate degradation of extracellular matrix proteins and proteolytic activation of chemokines [38,66].

**Table 4:** Classification of cytokines based on their most important functions in human AML; a summary of the classification used in previous clinical studies of systemic cytokine/chemokine profiles before and following intensive antileukemic treatment [67,68,69].

Cytokine classification	Cytokines
Chemokines	The CCL family of chemokines, 28 members numbered from CCL1 to CCL28
	The CXCL family of chemokines, 16 members numbered from CXCL1 to CXCL16 (including CXCL8 that is also referred to as IL8)
	C (γ) chemokines: XCL1, XCL2
	CX3CL1
Interleukins	The major immunoregulatory interleukins, including IL1, IL2, IL4, IL5, IL6, IL7, IL8, IL9, IL10, IL11, IL12, IL13, IL17
	IL1 receptor antagonist (a natural receptor antagonist)
Growth factors	IL3
	Granulocyte-macrophage colony-stimulating factor (GM-CSF), granulocyte colony-stimulating factor G-CSF,
	macrophage colony-stimulating factor (M-CSF), fms-like tyrosine kinase ligand (Flt3 L)
	Vascular endothelial growth factor (VEGF, hepatocyte growth factor (HGF), basic fibroblast growth factor (bFGF)
	epithelial growth factor (EGF9
	Erythropoietin (Epo), thrombopoietin (Tpo), stem cell factor (SCF)
	Leptin
Immunoregulatory cytokines	CD40 Ligand, Interferon ( IFN)γ, tumor necrosis factor (TNF)α

## 4. Methodological Strategies for Analysis of Cytokine Profiles

### 4.1. Serum *versus* Plasma Samples

Serum samples are prepared after *in vitro* coagulation, and during this *ex vivo* handling, the platelets are activated and release soluble mediators, including several chemokines [73]. The cytokine profiles in serum and plasma will therefore differ due to this *ex vivo* platelet activation. Despite this, serum samples have been used for prognostication in AML [40], and for several mediators, the contribution from *ex vivo* platelet release seems to be relatively small compared to the *in vivo* variations. 

Previously established biobanks may only include serum samples; if so, one has to consider whether altered serum levels of a platelet-released mediator reflect *in vivo* processes or different peripheral blood platelet counts, leading to differences in *ex vivo* release during sample preparation. Different approaches can then be used for interpretation of results. Firstly, if platelet counts are available, one can evaluate whether mediator serum levels are correlated with the platelet counts. Secondly, a correlation map or hierarchical cluster analysis can be made for different platelet-released mediators to see whether they correlate with each others. Finally, if different platelet-expressed mediators show qualitatively different alterations (increased *versus* decreased), this cannot be explained by a platelet-dependent effect. The best solution will of course be simply to use plasma instead of serum samples if platelet-released mediators are to be investigated. However, platelet levels of various soluble mediators show a wide variation, and future studies should clarify which platelet mediators that are released at low levels during serum sample preparation and, thereby, do not make a significant contribution to the serum levels. Whether there are differences between various plasma samples (heparin *versus* ethylenediaminetetraacetic acid (EDTA) *versus* citric acid as anticoagulants) should also be examined. 

### 4.2. Design of Normal Control Groups

As described above and summarized in Table 2, certain cytokines show age-dependent variations in their serum levels in patients and/or healthy controls. It is therefore essential that the control groups of healthy individuals are carefully matched with the patients when comparing serum levels in healthy individuals and patients. The characteristics of the control groups are not described in detail in several of the previous studies summarized in Table 2, and differences between studies with regard to matching of the controls may explain at least some of the conflicting results. 

### 4.3. Analysis of Cytokine Profiles—Statistics and Bioinformatics

The handling of the overall data from systemic cytokine profiles, including the levels of 30–50 mediators per sample, requires the use of bioinformatical tools for analysis, e.g., hierarchical clustering or principal component analyses to identify patient subsets or cytokine clusters [74,75]. The methodologies for clustering and other bioinformatical analyses of protein levels are in principal the same as for analysis of global gene expression analyses [74,76] that are used for subclassification of cancer patients, including AML patients [76,77,78]. Similar analytical strategies can thus be used in such proteomic studies [79,80,81], and such analysis of systemic serum/plasma protein profiles is helpful when trying to identify biomarkers to be used in diagnostic or prognostic evaluation [40,53,68,82]. It is not trivial to establish a threshold or convert them into decision making tools, and incorporation into routine clinical practice is not straight forward. However, in clinical studies, they can be used to identify the most useful markers [68,80,82]. An example of this is the possible use of chemokine levels in the diagnosis of myelodysplastic syndromes, where the bioinformatical analysis identified CCL3 and Tpo as the most useful markers (see below).

## 5. Effects of Locally Released Soluble Mediators on Distant Organs—The Lesson from Mesenchymal Stem/Stromal Cell Infusions

Mesenchymal stem cells have now been established as important immunomodulatory cells, with an ability to promote repair of injured tissue [83,84]. Similar cells are also present in tumors and may then be important for immunoediting during disease development [85]. Infusion of these cells is now tried for immunomodulation [86,87]; the cells then mediate their effects through the release of soluble mediators, as well as through interactions with Treg and Th17 cells [88,89,90,91]. The infused mesenchymal cells are mainly trapped in the lungs, and their immunosuppressive activity in distant organs is mediated through their release of soluble mediators [83,86,87,92,93]. Such distant effects are probably important for the beneficial effect of such therapy in patients treated with allogeneic stem cell transplantation and developing severe posttransplant graft *versus* host disease (GVHD) mainly affecting the skin, liver and gastrointestinal tract [86,87]. However, mesenchymal stem cells also release growth factors that can be important in the regulation of both angiogenesis and cancer cell survival [94,95,96]. Mesenchymal stem cells can even release soluble mediators that (i) support cancer cell survival and contribute to chemoresistance in response to conventional chemotherapy [97] or (ii) soluble mediators, which inhibit malignant cell growth [94]. This potential role of mesenchymal stem cells as delivery vehicles for proteins with pro- or anti-tumor properties is considered a potential target for future intervention in cancer therapy [95]. In the present context, the distant effects of mesenchymal stem cells that are trapped in the lungs clearly illustrate that the systemic levels of chemokines/cytokines can mediate important biological effects.

Mesenchymal stem cells have also been suggested as delivery vehicles for anticancer proteins [94,95]. The biological basis for this suggestion is that stromal cells are home to tumors, where they contribute to the tumor-associated stroma. Infused mesenchymal stem cells are mainly trapped in the lungs, and studies in an experimental animal model of pulmonary metastasis showed that infusion of stromal cells engineered to release anticancer interferon-β (IF-β) then caused regression of metastases [94]. An alternative methodological approach for extrapulmonal tumors could be direct injections of engineered stromal cells. If such a therapeutic strategy should be used in hematologic malignancies that show diffuse infiltration throughout the bone marrow compartment, one would have to use cells that were engineered not only to release anticancer proteins; the cells would also need to avoid pulmonary trapping and instead show specific homing to the bone marrow compartment. If so, one possible approach could be to use bone marrow stromal cells engineered to release T-cell chemotactic chemokines that could increase local recruitment of antileukemic T-cells to the bone marrow microenvironment and, thereby, reduce the risk of relapse after allogeneic stem cell transplantation. 

## 6. Systemic Cytokine Profiles in Clinical Hematology

### 6.1. Similarities between Normal and Pathological Cytokine Profiles

Cytokine/chemokine profiles can be altered by diseases, as well as therapeutic interventions, but despite this, there are certain common characteristics of the profiles for patients (e.g., allogeneic stem cell transplant recipients, autotransplanted patients and patients with untreated AML, MDS or multiple myeloma) and healthy individuals [40,53,68,98,99], the most important being that chemokine levels are generally high, whereas interleukin levels are relatively low or undetectable. The systemic levels of other immunoregulatory cytokines show considerable variation. The growth factor levels also vary between patients, even though G-CSF levels are generally relatively high, whereas Tpo levels are usually inversely correlated to platelet production [59]. 

### 6.2. Prognostication of Patients Receiving Conventional Intensive AML Chemotherapy

Kornblau *et al*. [40] investigated serum cytokine profiles as a possible prognostic parameter for AML patients receiving intensive chemotherapy. They examined 27 cytokines (including the chemokines CCL2/3/4/5/11, CXCL8/10). Kornblau *et al*. first investigated the associations between prognosis and the serum levels of individual cytokines, and patients who attained remission were more likely to have (i) increased levels of CCL5, as well as IL2/4/5/10, and (ii) decreased levels of CCL2 and TNFα. Furthermore, long-time survival was only associated with increased levels of CCL5 and IL2/5, as well as decreased levels of CCL4 and CXCL8. Thus, mainly chemokines showed significant associations when investigating serum levels of individual cytokines. 

As described above, Kornblau *et al*. [40] investigated 27 cytokines, and 11 of them separated the samples into at least two different normally distributed classes; among these were CCL3 and CCL5. Hierarchical clustering based on these 11 bimodal cytokines identified eight patient clusters that could be classified as favorable (three clusters), intermediate (three clusters) and unfavorable (the two last clusters) with regard to significant differences in remission rate and median survival (52 *vs.* 32 *vs*. 16 weeks, *p* = 0.003). Even though the serum cytokine profile can be used for prognostic classification of patients, it is difficult to see how such complicated bioinformatical analyses can be transformed into parameters suitable for routine clinical practice and prognostic evaluation of individual patients. Rather, the practical clinical use will probably depend on the identification of a limited number of key mediators. Certain cytokine levels were also correlated with cytogenetic abnormalities; an observation supporting our previous conclusion that it can be difficult to use single cytokine levels as independent clinical parameters in AML. 

### 6.3. The Cytokine Profiles in AML Patients Receiving Low-Toxicity Disease-Stabilizing Treatment Based on Valproic Acid and All-Trans Retinoic Acid (ATRA)

Disease-stabilizing low-toxicity treatment is now tried in AML, and a recent study investigated the cytokine profiles in patients receiving valproic acid + ATRA (including the chemokines CCL2/3/4/5/11 and CXCL5/8/10/11) [39]. The systemic cytokine profile is also altered by treatment with valproic acid, all-trans retinoic acid or low-toxicity chemotherapy, but the effects differ between patients and cannot be used to predict response to treatment. 

### 6.4. Chemokine Serum Levels in Patients Receiving Disease-Stabilizing Treatment with Azacitidine Alone or Sequential Azacitidine and Lenalidomide

A recent study investigated the effects of azacitidine alone or sequential azacitidine plus lenalidomide in elderly AML patients, including effects on serum cytokine levels [100]. These patients also had relatively short response duration of 6.2 months; this is comparable to patients treated with valproic acid plus all-trans retinoic acid (ATRA). Decreased pretreatment levels of five cytokines were significantly associated with later response to treatment (including CXCL9), whereas nine cytokines (including CCL3 and CXCL5) showed increased levels after treatment, independent of the response to treatment (including CCL3 and CXCL5). Thus, the predictive value of pretreatment chemokine levels and the effects of treatment on systemic chemokine levels/profiles differ between various alternatives for low-intensive disease-stabilizing therapy (valproic acid + ATRA *versus* azacitidine + lenalidomide). 

### 6.5. Systemic Chemokine Levels in the Preleukemic MDS

Recent studies suggest that chemokine expression levels have a prognostic impact in MDS. Results from analyses of CCL2, CCL3, CCL4, CCL5, CCL11, CXCL8 and CXCL10 in serum for 117 MDS patients showed that the mean CCL3 level was significantly lower in MDS compared with normal samples, and the CCL5 levels also seemed to be lower in MDS. In contrast, the mean expression of CXCL8 and CXCL10 was significantly higher than normal in MDS. The CCL2, CCL4 and CCL11 levels were not statistically different in MDS compared with the normal controls. Relatively high CCL3 levels were associated with longer survival in MDS. Finally, the levels of these chemokines did not differ between AML and MDS patients, except for CXCL8 that was higher in AML [40]. 

Increased levels of CXCL8 in MDS-patients were also detected in another study investigating serum levels in MDS patients with thrombocytopenia, and similar to AML, the CXCL8-levels then decreased to normal values during cytoreductive treatment and achievement of complete remissions [47]. CXCL4 and CXCL7 serum levels are decreased in MDS patients [101]; although this decrease was more pronounced in patients with advanced disease, it was also detectable in early stage MDS and may therefore be of diagnostic relevance. Kordasti and coworkers compared immunological features in low-risk (*i.e*., mainly the risk of AML transformation) *versus* high-risk MDS and found serum levels of CCL5 to be significantly increased in low-risk MDS [102]. Thus, MDS patients show a specific systemic chemokine profile that at least partially overlaps with the AML profile (CCL2/3/4/5, CXCL8/10; see Table 2) and the use of chemokine profiling may be relevant for the prognostic classification of these patients. 

### 6.6. Systemic Cytokine Profiles as a Diagnostic Tool in Preleukemic MDS

The generally accepted classification of hematologic malignancies published by the World Health Organization (WHO) gives detailed guidelines with regard to diagnostic criteria for hematologic malignancies [103]. However, in certain patients, it may still be difficult to distinguish between different diagnostic possibilities. One such diagnostic challenge can be to distinguish hypoplastic MDS from aplastic anemia based on morphology alone when cytogenetic analyses are normal. The possible use of serum cytokine profiles (including the chemokines CCL2/3/4/5/11 and CXCL5/8/10/11) as a diagnostic tool was investigated by Feng *et al.* [53]; different profiles were then detected for MDS and aplastic anemia, and Tpo, together with CCL3 levels, it was particularly important to distinguish between the two. Aplastic anemia was characterized by high Tpo and normal CCL3 levels, whereas MDS patients showed normal Tpo and increased CCL3 levels. In this case, the bioinformatical analysis could be used to identify two key cytokines (including one chemokine) that may become a part of future clinical practice if the observations can be confirmed by others. 

### 6.7. The Pretransplant Cytokine Profile as a Possible Risk Factor for Acute GVHD in Patients Receiving Allogeneic Stem Cell Transplantation

Previous studies have demonstrated that the risk of acute GVHD in allotransplanted patients is associated with certain pretransplant factors, including age, the extent of previous chemotherapy and the conditioning regimen [104,105]. In a recent study, we investigated whether the pretransplant serum cytokine profile (including the chemokines CCL2/3/4/5/11 and CXCL5/8/10/11) was associated with the development of serious posttransplant complications, *i.e.*, early multiorgan failure or severe acute GVHD [68]. We investigated the levels of 33 cytokines, including several chemokines, in 44 consecutive allotransplant recipients, and we identified a group of patients with a specific cytokine profile and a low frequency of early posttransplant complications. This subset was characterized by altered levels of several soluble mediators, and especially by increased levels of the potentially immunosuppressive mediators, G-CSF, HGF, IL1 receptor antagonist (IL1RA) and tumor necrosis factor receptor-1 (TNFR1). CCL2 was the only chemokine that contributed significantly to the identification of this patient subset. 

We described that high pretransplant levels of HGF are associated with an increased risk of GVHD [75], whereas the posttransplant development of acute GVHD is associated with an increased risk of acute GVHD (see the detailed discussion below). This difference may be explained by the diverse biological effects of HGF; this cytokine is an important regulator of angiogenesis, as well as immune responses, and the different impact of increased pre- and post-transplant serum levels may reflect predominating effects on different biological processes previous to (e.g., T-cells and dendritic cells) and following (e.g., endothelial cells, GVHD-associated endothelial cell damage or angiogenesis) allogeneic stem cell transplantation [35,38,106,107,108,109].

### 6.8. Serum Cytokine Levels to Diagnose and Predict Outcome in Acute GVHD

Several previous studies have investigated the possible use of serum cytokine levels to diagnose or predict treatment outcome in acute GVHD [110]. The strategy for identifying biomarkers in human GVHD is summarized in Table 5. For several cytokines, the results are conflicting, an observation supporting our previous statement that variations in single cytokine levels are difficult to use in routine patient handling. 

**Table 5:** Systemic cytokine levels and cytokine profiles as biomarkers of acute graft *versus* host disease (GVHD); the way from studies of single cytokines to the description of a soluble mediator profile [82,110,111,112,113,114].

**1. Studies of single cytokines in acute GVHD**	
**Acute GVHD is associated with increased systemic levels of single proinflammatory cytokines; for references, see [110]**	
IL6, IL8/CXCL8	Both increased
IL12	Divergent effects; most studies describe normal levels, but one study described increased levels
IL15, IL18	Both increased
TNFα	Divergent results; this cytokine has been investigated in several studies and both increased and normal levels have been described
TNF receptor 1	Increased
IL2 receptor	Divergent effects; most studies describe increased levels, but normal levels were described in one study
IFNγ	Divergent effects; most studies described increased levels, but one study described normal levels
HGF	Increased
**2. Analysis of a large panel of immunoregulatory soluble mediators and selection of markers for further studies.**	
**A study of systemic levels of 120 mediators in allotransplanted patients with acute GVHD, including the chemokines CCL2, CCL3, CCL5, CCL7, CCL8, CCL11, CCL13 and CXCL10 together with other cytokines, soluble receptors and adhesion molecules [82]. Four markers of particular importance were identified as markers of acute GVHD.**	
IL8/CXCL8	Important for local recruitment of immunocompetent cells; additional proangiogenic effects
IL2 receptor γ	Activated T-cells show increased expression of this growth factor receptor
HGF	An immunoregulatory cytokine that may have immunosuppressive effects, but shows increased systemic levels in human acute GVHD
TNFR1	TNFα is a proinflammatory cytokine released by many immunocompetent cells
**3. Addition of organ-specific markers.**	
**Acute GVHD is seen especially in the skin, liver and gastrointestinal tract [112,113,114]. Two organ-specific markers were added to the immunoregulatory markers** .	
Elafin	A skin-specific marker
Reg-3α	This marker is expressed especially in the gastrointestinal tract
**4. Validation of a simplified systemic soluble mediator profile for diagnosis and prognostication in acute GVHD [114].**	
Conclusion: A simplified systemic profile consisting of four immunoregulatory mediators (including the CXCL8 chemokine) and two organ-specific markers can be used for early diagnosis and prognostication of acute GVHD.	

Paczesny *et al*. [82] used a methodological strategy where they investigated the serum levels of 120 mediators, including the chemokines CCL2, CCL3, CCL5, CCL7, CCL8, CCL11, CCL13 and CXCL10. Their study also included angioregulatory and immunoregulatory cytokines, soluble adhesion molecules, hematopoietic growth factors, MMPs and protease inhibitors. Thus, they investigated systemic chemokine levels as a part of an extended soluble mediator profile. Based on their training set of 42 patients, they identified eight potential biomarkers for the diagnosis of acute GVHD, and additional studies in 424 patients demonstrated that the four mediators CXCL8, IL2 receptor α (IL2Rα), TNFR1 and HGF, optimally discriminated patients with and without acute GVHD. The three most important target organs for acute GVHD are the skin, liver and gastrointestinal tract; later studies showed that the two organ-specific molecules, (i) elafin as a marker of acute skin GVHD [111] and (ii) regenerating islet-derived 3-α (Reg-3α) [112,113], as markers of gastrointestinal affection could be used together with the four inflammatory immunoregulatory markers to diagnose acute GVHD. 

The four markers, CXCL8, IL2R-α, TNFR1 and HGF, identified above showed increased levels in acute GVHD, and increased levels of these markers have also been identified in other clinical studies [115]. These mediators may not only be important as diagnostic and prognostic markers of acute GVHD, they may also represent possible therapeutic targets in the treatment of this posttransplant complication. Firstly, chemokine receptors are now being developed, including inhibitors of the two receptors, CXCR1 and CXCR2, that show approximately 78% sequence identity and bind CXCL8 [116,117]. CXCL8 is important, both for development of angiogenesis and for T-cell chemotaxis [42,117]; CXCR1/CXCR2 inhibition may thus have several beneficial effects in these patients, including (i) inhibition of GVHD associated angiogenesis; (ii) inhibition of T-cell recruitment to GVHD-affected organs and (iii) possibly an antileukemic effect with reduction of posttransplant relapse risk through inhibition of local angiogenesis induced by residual leukemia cells. Secondly, monoclonal antibodies directed against the IL2 receptor (CD25) are now available; several prospective studies of anti-CD25 treatment for acute GVHD have been published, and this therapeutic strategy can be effective in steroid-refractory GVHD [118]. Thirdly, several TNF inhibitor are also available [119], and a recent study, including 97 patients, suggested that prophylactic use of the TNF inhibitor, etanercept, can reduce the incidence and severity of acute GVHD [120]. Finally, several strategies for inhibition of HGF or HGF-induced intracellular signaling are currently being developed [121,122], but to the best of our knowledge, this strategy has not been investigated in clinical trials for patients with acute GVHD. Taken together, these observations clearly illustrate that the use of systemic cytokine profiles may not only be useful for diagnostication and prognostication, but may also identify new possible therapeutic strategies. 

Levine *et al*. [114] evaluated whether the six mediators identified above (CXCL8 being the only chemokine) could predict treatment outcome in acute GVHD. They measured the serum levels of these markers at the time when GVHD treatment was started and later 14 and 28 days after the start of treatment, and they concluded that this biomarker panel measured at the time of acute GVHD diagnosis predicted both for day 28 non-responsiveness to treatment and mortality after 180 days. With regard to the increased CXCL8 levels, this study showed that (i) CXCL8 levels at the time of acute GVHD diagnosis were significantly correlated only with TNFR1, Reg-3α and HGF; (ii) in univariate analysis, CXCL8 levels at the time of GVHD diagnosis showed a significant correlation to the day 14 treatment response, but not to the day 28 response and (iii) CXCL8 was the only single mediator showing a significant correlation with day 180 mortality at all three time points tested (days 0, 14 and 28) in univariate analysis and showing a significant correlation with day 180 mortality already at the time of diagnosis (*p* = 0.025). One reason for this particular importance of CXCL8 could be that it is important both in inflammation and as a proangiogenic chemokine that may be involved in the neovascularization known to take place in acute GVHD [105]. 

Taken together, these observations support our previous hypothesis that that the most likely clinical use of systemic serum/plasma chemokine levels will be as parts of biomarker panels, rather than use as single markers. This may be due to the fact that most chemokines are released by a wide range of cells and organs, usually act on many different cells and often bind to different receptors (Table 1). This lack of specificity may then require that their clinical use is combined with organ-specific markers, as illustrated above, because one would expect chemokine levels mainly to reflect the nature (e.g., inflammation, angioregulation) and strength of an ongoing process, rather than the localization. 

### 6.9. The Cytokine Profile Late after Allogeneic Stem Cell Transplantation

We have investigated the systemic cytokine profile (33 cytokines examined) 3–6 months after allogeneic stem cell transplantation [68]. Even for patients without chronic GVHD, we detected abnormal profiles compared with healthy individuals, an observation suggesting that these patient profiles reflect that hematopoietic, and especially, immunological reconstitution is still not completed [123]. We could not identify a specific profile for patients developing chronic GVHD either, but these observations should be interpreted with great care, because our study was relatively small. 

## 7. The Importance of Sampling Standardization When Analyzing Effects of Therapeutic Interventions

The systemic cytokine/chemokine profiles can also be altered by clinical interventions. Firstly, platelet transfusions will cause an alteration of the systemic cytokine profile with an increase especially in platelet-derived cytokines [98]. Secondly, the systemic profile, and especially the chemokine levels are also altered by autologous stem cell harvesting [99]; but, alterations induced by platelet transfusions and stem cell harvesting will often last for less than 24 hours. Thirdly, intensive chemotherapy, febrile neutropenia and post-chemotherapy regeneration will alter the levels of several cytokines, soluble adhesion molecules and soluble cytokine receptors [49,124,125,126]. Finally, diurnal alterations [127,128] and age [44], may also influence systemic cytokine levels. Taken together, these observations clearly illustrate that the clinical context and sampling standardization is important when analyzing overall systemic cytokine profiles. 

## 8. Concluding Comments

Several recent studies suggest that the analysis of systemic cytokine profiles (including chemokine levels) can be used to identify biomarkers that are useful in routine clinical practice. However, the available hematological experience clearly illustrates that future clinical studies have to be carefully designed, and the following aspects have to be considered.

- Platelets contain a wide range of chemokines that can be released during activation, including CCL2, CCL3, CCL5, CCL7, CCL17, CXCL1, CXCL4, CXCL5, CXCL7, CXCL8/IL8 and CXCL12 [73]. Platelet release during preparation of serum samples will influence these levels, and plasma samples may therefore be more convenient when these mediators are analyzed.
- Systemic plasma or serum cytokine profiles can be altered by several clinical procedures (e.g., transfusions, age, chemotherapy) and even diurnal variations; a careful standardization of sampling is therefore necessary.
- Chemokines should be included in evaluation of systemic cytokine profiles, because they are important for many different biological functions, and their levels, therefore, seem to reflect the nature (inflammation, platelet/endothelium activation, immune activation, angioregulation, altered hematopoiesis, platelet/endothelium interactions) and strength of the biological response, rather than the localization/organ involvement.
- Chemokines are released by a wide range of cells and in a wide range of organs, and the optimal clinical use of systemic chemokine analyses will probably require analyses of chemokines together with (i) organ-specific mediators and (ii) other soluble mediators that interact or contribute together with the chemokines in normal or pathological processes.

Despite these challenges with regard to standardization and limitations with regard to localization of pathological processes, our conclusion is that the clinical use of systemic cytokine/chemokine profiles should be further investigated. The hematological experience clearly suggests that such strategies can be used to identify diagnostic and prognostic markers, especially when analyses of chemokine levels are combined with the analysis of other soluble mediators.
